# Underwater Target Tracking Using Forward-Looking Sonar for Autonomous Underwater Vehicles

**DOI:** 10.3390/s20010102

**Published:** 2019-12-23

**Authors:** Tiedong Zhang, Shuwei Liu, Xiao He, Hai Huang, Kangda Hao

**Affiliations:** 1 National Key Laboratory of Science and Technology on Underwater Vehicle, Harbin Engineering University, Harbin 150001, China; zhangtiedong@hrbeu.edu.cn (T.Z.);; 2 School of Materials Science and Engineering, Tianjin University, Tianjin 300072, China

**Keywords:** AUV, underwater target tracking, Gaussian particle filter, adaptive fusion strategy

## Abstract

In the scenario where autonomous underwater vehicles (AUVs) carry out tasks, it is necessary to reliably estimate underwater-moving-target positioning. While cameras often give low-precision visibility in a limited field of view, the forward-looking sonar is still an attractive method for underwater sensing, which is especially effective for long-range tracking. This paper describes an online processing framework based on forward-looking-sonar (FLS) images, and presents a novel tracking approach based on a Gaussian particle filter (GPF) to resolve persistent multiple-target tracking in cluttered environments. First, the character of acoustic-vision images is considered, and methods of median filtering and region-growing segmentation were modified to improve image-processing results. Second, a generalized regression neural network was adopted to evaluate multiple features of target regions, and a representation of feature subsets was created to improve tracking performance. Thus, an adaptive fusion strategy is introduced to integrate feature cues into the observation model, and the complete procedure of underwater target tracking based on GPF is displayed. Results obtained on a real acoustic-vision AUV platform during sea trials are shown and discussed. These showed that the proposed method is feasible and effective in tracking targets in complex underwater environments.

## 1. Introduction

After decades of research and development, autonomous underwater vehicles (AUVs) are becoming accepted by an increasing number of users in various military and civilian establishments. AUVs globally sold to customers are becoming progressively sophisticated through improvement of their self-governance capabilities, which allows them to deal with increasingly complex missions [1,2,3,4,5]. When AUVs move in unknown marine environments, a relative motion state appears between targets in the scene and AUVs. Thus, it is greatly significant for AUV autonomy to enhance moving-target prediction ability under complex dynamic backgrounds by using human perception [6,7,8].

As a particularity of underwater environments, acoustic vision is still a useful means of long-distance measurement for AUVs, so it is an important issue to understand the moving status of underwater targets on the basis of acoustic-vision information. At present, some significant achievements have been obtained. Williams [9,10] used temporal feature measures to provide a quantitative description of a moving target’s behavior over several scans, which was verified by a diver tracking experiment. Furthermore, he discussed [11] a tracking method of underwater targets based on optical-flow theory, and a tracking tree was constructed storing tracking information to enhance robustness. Chantler [12] and Ruiz [13] presented different approaches for classification and obstacle tracking, and the robustness of an interframe feature-measurement classifier for underwater-sector sonar scan images was examined. Perry [14,15] proposed a detection method of underwater targets based on machine-learning techniques. The self-learning function of neural networks was used to analyze the feature variation of acoustic images, and target regions were effectively distinguished. Williams [16] proposed a tracking method based on Kalman filters. Multiple targets were differentiated by clustering sonar returns, and Kalman filters were then used to track both stationary and moving obstacles. DeMarco [17] discussed diver detection and tracking by high-frequency forward-looking-sonar (FLS). Cluster classification was accomplished by matching observed cluster trajectories with trained hidden Markov models. Results showed that the diver could be autonomously distinguished from stationary targets in a noisy sonar image. Petillot [18] proposed a tracker based on a combination of segmentation and object-based feature extraction, and a nearest-neighbor algorithm was adopted to match detected targets and tracking-process accuracy and robustness based on the extended Kalman Filter (EKF) were improved. Clark [19,20] proposed an underwater-target-tracking method based on a probability-hypothesis density filter, and the predicted target position was fused with trajectory data. Experiments proved that tracking stability was better than that of Lalman filters. Handegard [21] presented automatic tracking of fish populations using FLS in which the automatic tracker was evaluated using three test datasets with different target sizes, observation ranges, and densities. Ma [22] proposed a single-target tracking method of noncomplexity backgrounds by using a particle filter (PF) and the correlation-matching method. Liu [23] proposed a target-tracking method based on variable image templates. Target features were obtained by surface let transform, and a particle filter was used to estimate the moving state of targets. Quidu [24] used statistical deviations in small patches of acoustic-vision-sequence information to detect targets in front of AUVs, and experiment results were in agreement with theoretical analysis. Natàlia Hurtós [25] presented two detectors based on FLS data and multibeam data, and they were combined with adequate planning and control strategies to detect, follow, and map an underwater chain. AI Muallim [26] proposed a robust wake-detection algorithm to improve divers tracking in acoustic vision, and the Kalman tracker was fine-tuned, attaining stable diver tracks in the test. Ye [27] proposed a moving-target-tracking method based on FLS, and a five-layer siamese network was designed to achieve a good tracking result. Results showed that it improved tracking accuracy and real-time performance.

Although many tracking methods were proposed to resolve the acoustic-vision tracking problem of multimoving targets under dynamic backgrounds, some problems need to be studied further. First, the mentioned methods are often unable to cope with significant appearance changes. In this scenario, it was shown that the features of moving targets (such as intensity and shape) in acoustic vision are often different in two successive times in a shift of relative distance, relative orientation, and relative attitude between moving targets and AUV. These challenges are particularly difficult for the mentioned methods when there are limited stable characteristics about targets of interest in acoustic images. Second, a target presents nonlinear motion relative to AUV. Some mentioned methods can resolve the tracking problem under these conditions, but their models often involve many parameters that must be tuned to obtain good performance (e.g., “forgetting factors” that control how fast the appearance of the model can change, and “resampling strategies” that control resampling quality and computational complexity), and can suffer from drift problems when an object undergoes partial occlusion. No solution regarding the feature description of moving targets or the feature set of targets in acoustic images has been proposed, and no optimization target-tracking strategy based on forward-looking sonar has been suggested for the complete multimoving-target-tracking procedure. On the basis of the above achievements, this paper establishes an online processing framework based on acoustic-vision images, and a novel method based on Gaussian PF (GPF) is presented. The hardware architecture and software architecture are summarily introduced, and the image characters of an underwater acoustic image are analyzed. The image-preprocessing method is discussed, and modified means of a median filter and region-growing segmentation are proposed to obtain regional information, based on which some suitable features are selected by a generalized regression neural network (GRNN). Thus, every subclass is characterized with a unique combination of these features, and multifeature adaptive fusion was designed for the measurement-model establishment of GPF. Then, the complete procedure of underwater-target tracking based on improved GPF is displayed. Results showed that the presented method avoids the resampling step. The particle-degeneracy phenomenon was compared with PF and it satisfied robustness and real-time properties. Its performance is superior to the EKF and PF in terms of accuracy, computational load, and other aspects, and it is a feasible and effective method for target tracking in complex underwater environments.

## 2. FLS Overview

**Figure 1:**
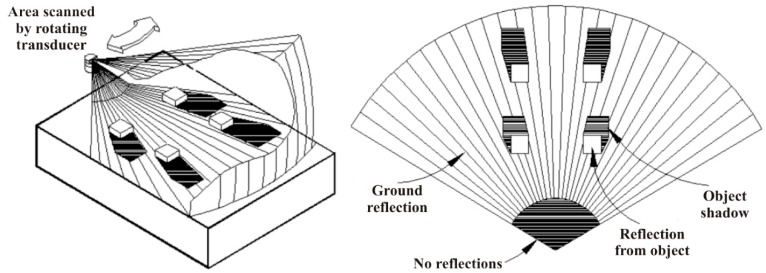
Diagram showing scanning procedure and idealization of expected return of used sonar [28].

The acoustic images were gained using Seaking DST Sonar, a type of FLS which is manufactured by Tritech [28]. The sonar is characterized by a fan-shaped beam that is rotated mechanically to create a spatial map of its surrounding area, and it produces a single ping at each angle and waits for the return before stepping to the following angle, continuing until the entire sector is scanned. Returns from each ping are then used to create the image, as is shown in Figure 1. It is the type of sonar most commonly used for collision avoidance, but also finds applications in mine detection and surveillance. Specifications of the sonar are shown in Table 1.

Acoustic images are formed by the echo intensity from the three-dimensional environmental space. Despite the wide-range advantage over standard vision, imaging sonar suffers from several drawbacks:

(1) The number of transducers that can be packed in an array is physically restricted because of the limitations of transducer size. Thus, the resolution of an FLS image is lower, and the gray level of the target area is generally smaller, so it is more difficult to find some details of targets inside it.
(2) The scattering capability of different parts of the target surface is different, which is affected by the shape, material, and relative position between target and sonar. The incident angle of an acoustic wave is also changed with target movement, so different regions may be generated for the same target in the acoustic image, and they often appear to be unconnected regions in acoustic vision.
(3) The phenomenon of multipath propagation is a distinctive feature in acoustic images, and reflected acoustic waves may have greater energy than that of ones reflected from obstacles, leading to false or lack of target detection, increasing the difficulty of acoustic-image processing.

**Figure 2:**
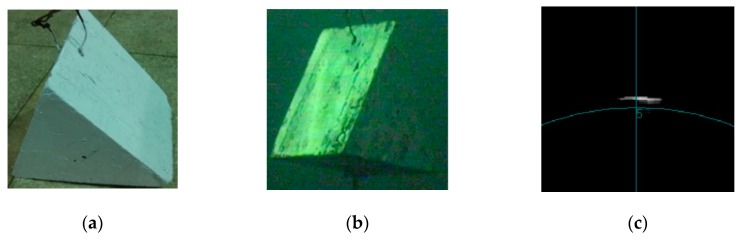
Image of target under different conditions: optical image gained in: (**a**) air and (**b**) water. (**c**) Acoustic image gained in water.

For the above, some images under different conditions are listed in Figure 2. It is shown that the characteristic of an acoustic image is different than those of optical images. Thus, some image-processing methods used in optical images have to be improved so that good results can be obtained.

## 3. Feature Selection Based on GRNN

The main goal of feature selection is to choose a number of features from the extracted feature set that yields minimum classification error. In this work, a feature-selection method based on a combination of GRNN and search procedures such as sequential forward selection (SFS) and sequential backward selection (SBS) was used to discover the optimal subset of features.

### 3.1. Feature Description 

It was supposed that the minimum size of outer rectangle of $R_{k}$ was m×n, $N^{o}$ the number of pixels of which $R_{k}$ consists, $N_{e}^{o}$ the number of pixels of which the edge of $R_{k}$ consists, $N^{b}$ the number of pixels of which the background region consists, S the number of intensity levels in the image, h(i,j) the element of second-order histogram H, $D_{e}^{o}\left(i\right)$ the Euclidean distances from point on the target’s perimeter curve to the target’s centroid, and $i=1,2,\ldots ,\;N_{e}^{o}$. Normalized central moments $\eta_{pq}$ of f(x,y) were defined to be:

(1) $$\eta_{pq}=\left(\sum_{i=1}^{m}\sum_{j=1}^{n}{\left(i-\bar{x}\right)}^{p}{\left(j-\bar{y}\right)}^{q}f\left(i,j\right)\right)/{\left[\sum_{i=1}^{m}\sum_{j=1}^{n}f\left(i,j\right)\right]}^{r}$$

where r=(p+q+2)/2 for p+q=2,3, …,∞, $\bar{x}=\sum_{i=1}^{m}\sum_{j=1}^{n}if\left(i,j\right)/\sum_{i=1}^{m}\sum_{j=1}^{n}f\left(i,j\right)$, and $\bar{y}=\sum_{i=1}^{m}\sum_{j=1}^{n}jf\left(i,j\right)/\sum_{i=1}^{m}\sum_{j=1}^{n}f\left(i,j\right)$.

Some possible features [29] were considered in this paper, which are described in Table 2.

### 3.2. Search Procedure

The SBS method performs a greedy space-searching technique. Starting by measuring performance on the original (unchanged) dataset, it proceeds by measuring classification performance by using classifiers that are induced in the datasets in which a single feature is omitted. Finally, the least significant feature is detected as the one that caused the lowest drop or highest gain in classifier performance [30]. This feature is afterwards omitted from the dataset, and the procedure is recursively repeated until the minimal required number of features remains or a certain stopping criterion is reached. The SBS procedure is as shown in Table 3.

In contrast to SBS, SFS starts with an empty data set and proceeds by expanding the data set with the feature, of which addition to the data set boosts the wrapped model performance most. The algorithm adds features in such manner recursively until the stopping criteria is met [31]. The procedure of SFS is as Table 4.

### 3.3. GRNN for Classification

**Figure 3:**
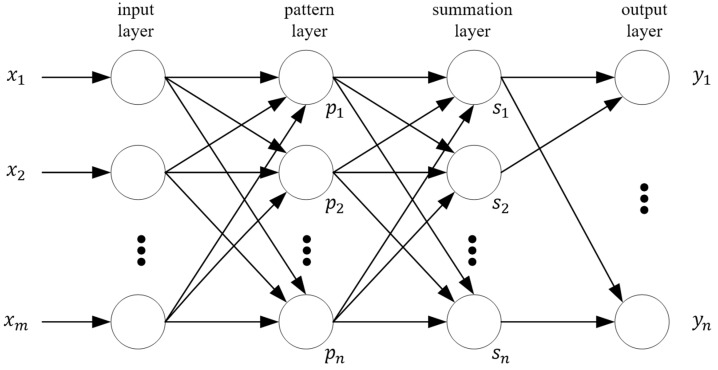
Generalized regression neural network (GRNN).

The GRNN that was proposed by Specht is a class of neural networks extensively used for function mapping between input and output variables [32,33,34,35], which is shown in Figure 3. It is a one-pass learning algorithm with a highly parallel network, and it does not require an iterative procedure. Thus, it provides fast training, and estimates can converge to the underlying (linear or nonlinear) regression surface even with sparse samples, that is, even with sparse data in a multidimensional measurement space, GRNN provides smooth transitions from one observed value to another, hence, it can be used for predicting, modelling, mapping, and interpolating continuous variables.

For the observed values X of random variable x, the regression of random variable y can be found using:

(2) $$E\left(y/X\right)=\int_{-\infty }^{+\infty }yf\left(X,y\right)dy/\int_{-\infty }^{+\infty }f\left(X,y\right)dy$$

where f(X,y) is a known joint continuous probability-density function.

When f(X,y) is unknown, it should be estimated from a set of observations of x and y. Probability estimator $\hat{f}\left(X,y\right)$ can be gained by the nonparametric consistent estimator suggested by Parzen as follows:

(3) $$\hat{f}\left(X,Y\right)=\frac{1}{{\left(2\pi \right)}^{(m+1)/2}\sigma^{m+1}n}\overset{n}{\underset{i=1}{\sum }}\exp[-\frac{{{(X-X}_{i})}^{T}{(X-X}_{i})}{{2\sigma }^{2}}-\frac{{\left(Y-Y_{i}\right)}^{2}}{{2\sigma }^{2}}]$$

where n is the number of observations, m the dimension of vector variable x, and *σ* the smoothing factor. $X_{i}$ and $Y_{i}$ are sample values of random variables x and y.

Substituting Equation (3) into Equation (2), the output $\hat{Y}(X)$ can be written as

(4) $$\hat{Y}(X)=\Sigma_{i=1}^{n}Y_{i}{\exp[-(X-X}_{i}{)}^{T}{(X-X}_{i})/{(2\sigma }^{2})]/\Sigma_{i=1}^{n}{\exp[-(X-X}_{i}{)}^{T}{(X-X}_{i})/{(2\sigma }^{2})]$$

### 3.4. Experiments and Analysis

**Figure 4:**
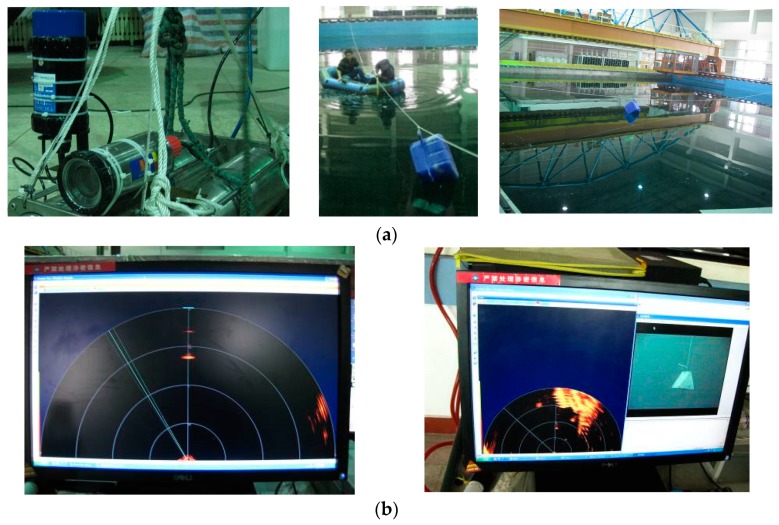
Experiment scene: (**a**) sonar and target launch and (**b**) collected data.

**Figure 5:**
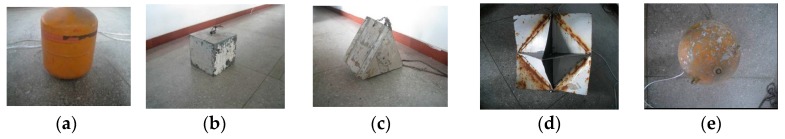
Selected experiment targets: (**a**) first target, pontoon; (**b**) second target, cube; (**c**) third target, triangular prism; (**d**) fourth target, reflector; and (**e**) fifth target, sphere.

**Figure 6:**
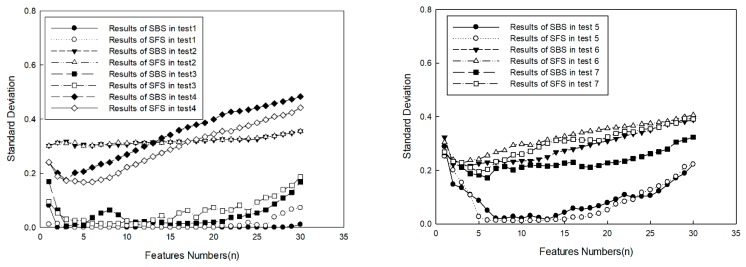

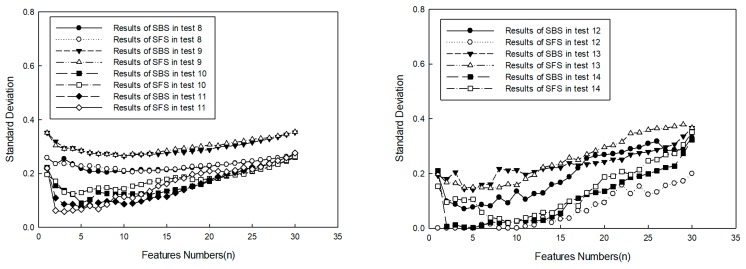
Classification error in tests.

**Figure 7:**
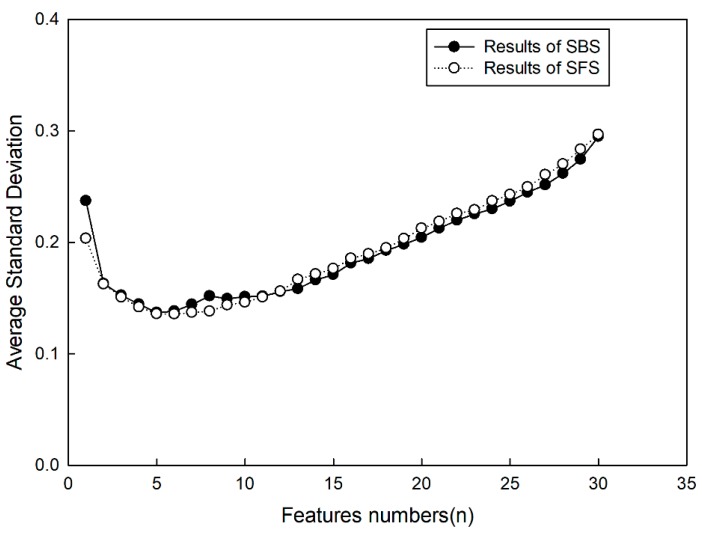
Average standard classification deviation.

Some experiments were carried out to obtain a representative subset of features in the tank, as shown in Figure 4. The targets consisted of a pontoon, a cube, a triangular prism, a reflector, and a sphere, which are shown in Figure 5 and Figure 6. A series of FLS images under different situations were obtained, as shown in Table 5. Results of feature selection obtained by SFS and SBS are shown in Figure 6 and Figure 7.

Figure 6 shows that, if statistic rules could not be founded by SFS and SBS in each test, then the average values of standard deviation were counted, which are shown in Figure 7. For the classification method, it was shown that average values gained by SFS were smaller than those gained by SBS if the number of selected features was less than 12. For selected features, it was shown that average values declined with the increase of selected features if the number of selected features was less than 5. This indicated some useful description information drawn into the classification by new added features of the target, so the accuracy of target classification was improved. In contrast, as the number of selected features was more than 5, errors increased with the increase of selected features. This indicated some useless description information draw into the classification by new added features, which had more of an effect on target classification, so error rate was raised. From the results, it can be seen that it was not beneficial for target classification to select more features. According to the results, it may have been the best choice to select five types of features, and for SFS to obtain the smaller classification error rate.

**Figure 8:**
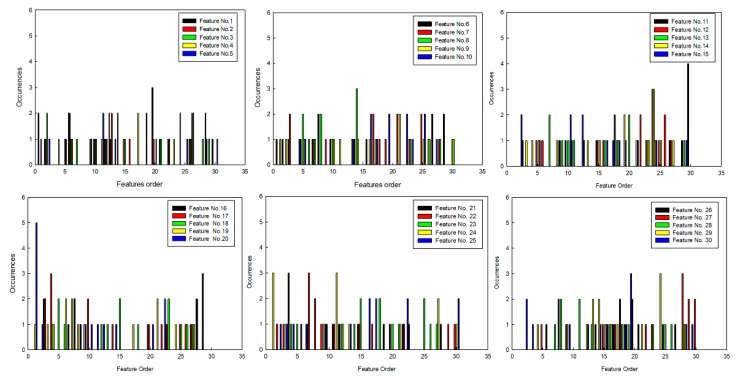
Statistical results.

**Figure 9:**
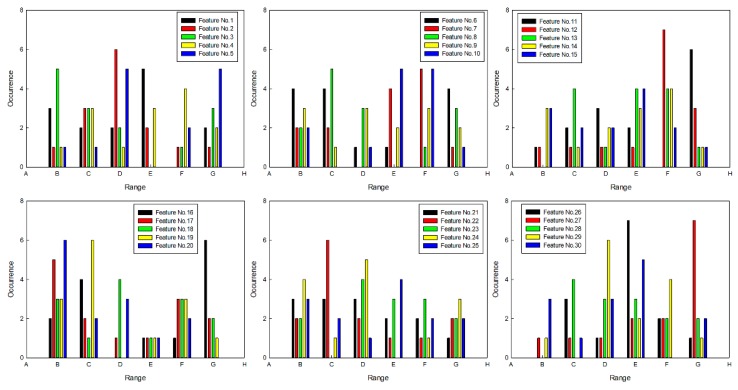
Statistical results.

On the basis of the above conclusions, the sets of features were selected by SFS, and the statistical results of feature order are shown in Figure 8. As only five types of features were used to set up the feature set, the feature order was divided into six intervals (shown in Table 6), and statistical results were rearranged, which are shown in Figure 9. Then, five types of features that had more occurrences were selected in interval B, and they are shown in Table 7.

## 4. Gaussian Particle Filtering

### 4.1. Basic Principle

GPF is a problem for traditional particle-filter resampling, and the Gaussian density function is used to approximate the posterior probability distribution of the state [36,37]. The density of Gaussian random variable x can be expressed as:

(5) $$N\left(x;\bar{x},\sigma \right)={\left(2\pi \right)}^{-m/2}{\left|\sigma \right|}^{-1/2}\exp[-\left(x-\bar{x}{)}^{T}\sigma^{-1}\left(x-\bar{x}\right)/2\right]$$

where x represents an m-dimensional vector, and $\bar{x}$ represents the mean of x. σ represents covariance.

As observation value $y_{t}$ at time t is obtained, the posterior probability distribution is approximated as:

(6) $$p\left(x_{t}|y_{0:t}\right)=C_{t}p\left(y_{t}|x_{t}\right)p\left(x_{t}|y_{0:t-1}\right)\approx C_{t}p\left(y_{t}|x_{t}\right)N\left(x_{t};{\bar{x}}_{t},{\bar{\sigma }}_{t}\right)$$

where $x_{t}$ represents the state value at time t; $y_{0:t}$ represents the set of observation sequence numbers from 0 to t, that is, $y_{0:t}=\left\{y_{0},y_{1},\ldots y_{t}\right\}$;$\;{\bar{x}}_{t}$ represents the mean of $x_{t}$; ${\bar{\sigma }}_{t}$ represents the mean of σ; and $C_{t}$ is a normalized constant, expressed as follows:

(7) $$C_{t}={\left(\int p(x_{t}|y_{0:t-1})p(y_{t}|x_{t})dx_{t}\right)}^{-1}$$

$p(x_{t}|y_{0:t-1})$ is prior probability distribution, and the GPF measurement update approximates the above prior probability distribution by Gaussian distribution $N\left(x_{t};{\bar{x}}_{t},{\bar{\sigma }}_{t}\right)$. Usually, the mean and covariance of $p(x_{t}|y_{0:t})$ are obtained by extracting K samples $x_{t,n}$(n=1,2,…,K) from importance function $q(x_{t}|y_{0:t})$.

Similarly, by approximating posterior probability distribution with Gaussian distribution function, the updated posterior probability distribution can be approximated as:

(8) $$p(x_{t}|y_{0:t})\approx N\left(x_{t};\mu_{t},\sigma_{t}\right)$$

As the measurement is updated, the GPF approximates predicted probability distribution $p(x_{t+1}|y_{0:t})$ to Gaussian distribution. Then:

(9) $$p\left(x_{t+1}|y_{0:t}\right)=\int p\left(x_{t+1}|x_{t}\right)N\left(x_{t};{\bar{x}}_{t},\sigma_{t}\right)dx_{i}=\frac{1}{K}\Sigma_{n=1}^{K}p(x_{t+1}|x_{t,n})$$

In the formula, particle $x_{t,n}$ is obtained by sampling $N\left(x_{t};{\bar{x}}_{t},\sigma_{t}\right)$. On the basis of observations at time t, by sequentially sampling state-transition distribution $p(x_{t+1}|x_{t,n})$ of n=1,2,…,K, state particle $x_{t+1,n}$ at time t+1 is obtained. Then, ${\bar{x}}_{t+1}$ and ${\bar{\sigma }}_{t+1}$ are calculated by the following formula:

(10) $${\bar{x}}_{t+1}=\frac{1}{K}\Sigma_{n=1}^{K}x_{t+1,n}$$

(11) $${\bar{\sigma }}_{t+1}=\frac{1}{K}\Sigma_{n=1}^{K}\left({\bar{x}}_{t+1}-x_{t+1,n}\right){\left({\bar{x}}_{t+1}-x_{t+1,n}\right)}^{H}$$

Then, the predicted probability distribution of the GPF can be approximated as:

(12) $$p\left(x_{t+1}|y_{0:t}\right)\approx N\left(x_{t+1};{\bar{x}}_{t+1},{\bar{\sigma }}_{t+1}\right)$$

### 4.2. Gaussian Particle-Filter Improvement

Although the particle filter provides a good probabilistic framework for target tracking, the target region lacks some details, such as those in optical images, and the information of region area, brightness, and contour is also unsteady, so it is difficult to track a moving target on the basis of only single-feature information in FLS image sequences. Thus, the method based on a feature set was used in this paper.

#### 4.2.1. Likelihood-Function Representation

For the feature set selected in Section 3.4, we supposed that the distribution of the *i*th feature at time t is expressed as $S_{t}^{i}={\left\{S_{t,n}^{i}\right\}}_{n=1,\ldots ,K}$, and the reference model of the *i*th feature is $S_{m}^{i}$. Then, the likelihood function based on the Gaussian model is written as [38]:

(13) $$P_{S}(y_{t}^{i}|x_{t})\propto \frac{1}{\sqrt{2\pi \alpha }}\exp\left(-\frac{\beta^{i}d^{2}\left(S_{t}^{i},S_{m}^{i}\right)}{2\alpha^{2}}\right)$$

where $y_{t}^{i}$ is the measurement of the i feature clue, α is the likelihood-function noise value, $\beta^{i}$ is the distance control coefficient, and $d\left(S_{t}^{i},S_{m}^{i}\right)$ refers to the distance between target template feature and each particle feature.

#### 4.2.2. Feature-Set Fusion Strategy 

Adaptive fusion (AF) is proposed to fuse the likelihood functions formed by the feature set, which can adaptively adjust fusion strategy according to the tracking situation. As a feature clue is good, multiplicative fusion (MF) is selected to obtain the likelihood function with higher confidence. Otherwise, it is switched to weighted fusion (WF), then, a more stable likelihood function is obtained.

WF is more stable for the problem of feature fusion under the interference condition, and its expression is as follows:

(14) $$p(y_{t}^{1},\ldots ,y_{t}^{m}|x_{t})=\sum_{i=1}^{m}a_{i}p(y_{t}^{i}|x_{t})$$

where $a_{i}$ is weighting coefficient of $p(y_{t}^{i}|x_{t})$ and $\sum_{i=1}^{n}a_{i}=1$.

On the basis of the independent assumptions of each feature, MF can achieve better tracking accuracy under less interference. The likelihood model for m feature multiplicative fusions is as follows:

(15) $$p(y_{t}^{1},\ldots ,y_{t}^{m}|x_{t})=\overset{m}{\underset{i=1}{\prod }}p(y_{t}^{i}|x_{t})$$

Considering the different advantages of WF and MF, the switch condition was set up on the basis of feature clues, which could be assessed by the covariance matrix. It was assumed that the dimension of $x_{t}$ is represented as *dim*, and the covariance of the *i*th feature is represented as $A_{i}$, then, it is written as:

(16) $$A_{i}=\Sigma_{n=1}^{K}p(y_{t}^{i}|x_{t,n})[x_{t,n}-\Sigma_{n=1}^{K}p(y_{t}^{i}|x_{t,n})x_{t,n}]{\left[x_{t,n}-\Sigma_{n=1}^{K}p(y_{t}^{i}|x_{t,n})x_{t,n}\right]}^{T}$$

and covariance matrix $\Delta_{i}$ is written as [39,40]:

(17) $$\Delta_{i}={\left(\Sigma_{a=1}^{dim}\Sigma_{b=1}^{dim}A_{a,b}^{2}\right)}^{1/2}$$

Threshold $T_{i}$ was set for each cue to determine whether the cue was degenerated. Then, the adaptive likelihood model could be written as:

(18) $$p\left(y_{t}^{1},\ldots ,y_{t}^{m}|x_{t}\right)=\{\begin{array}{c} \Pi_{i=1}^{m}p(y_{t}^{i}|x_{t}),\;1/\Delta_{i}>T_{i}\\ \sum_{i=1}^{m}a_{i}p\left(y_{t}^{i}|x_{t}\right),\;1/\Delta_{i}<T_{i} \end{array}$$

where $a_{i}$ is computed by the fuzzy logic method, and the algorithm is shown in Table 8.

#### 4.2.3. Target-Tracking steps

According to GPF theory, tracking-implementation steps based on FLS images are summarized as:

1. Initialization: to select interesting targets in first image frame. After the image is processed, target features in Table 7 are calculated, and the number of sample particles K is determined. It is assumed that the initial importance function is normal distribution function. Then, the mean value is the center coordinate $\left(x_{target},y_{target}\right)$ of the target, and covariance σ is determined by the tracking environment, that is, particles collected by the initial importance function in the x- and y-axes can be written as $N\left(x;x_{target},45\right),\;N\left(y;y_{target},40\right)$, and each particle is calculated according to the kinematics model.
2. To capture the image in the next frame, calculate features of particles ${\left\{x_{t,n}\right\}}_{n=1}^{K}$. According to Equation (17), feature clues are analyzed to check whether they are degenerated, and the fused weighted value of particles is calculated. The weighted particle value is normalized as $w_{t,n}=w_{t,n}/\sum_{n=1}^{K}w_{t,n}$; then, $\mu_{t}$ and $\sigma_{t}$ are calculated.
3. To sample according to posterior probability distribution $N\left(x_{t};\mu_{t},\sigma_{t}\right)$, and ${\left\{x_{t,n}\right\}}_{n=1}^{K}$ is gained. Then, $x_{t+1,n}$ can be calculated by the kinematics model. According to Equation (18), the predicted mean and covariance values are calculated. If targets are lost, covariance value is expanded, otherwise, it is turned into Step 2.

## 5. Example Test and Discussion

In order to evaluate the tracking method proposed in the paper, a series of tests were carried out in the tank and in the sea. In the tank experiment, it was compared with other methods in different motion scenarios, and its advantages were assessed. In the sea test, the method was downloaded to the AUV system, and its adaptability was assessed. Center position error (CLE) was used to calculate the error that was the Euclidean distance between tracked center position $\left(x_{p},y_{p}\right)$ and real center position $\left(x_{g},y_{g}\right)$. Its formula is:

(19) $$CLE=\sqrt{{\left(x_{p}-x_{g}\right)}^{2}+{\left(y_{p}-y_{g}\right)}^{2}}$$

### 5.1. Tank Experiment

**Figure 10:**
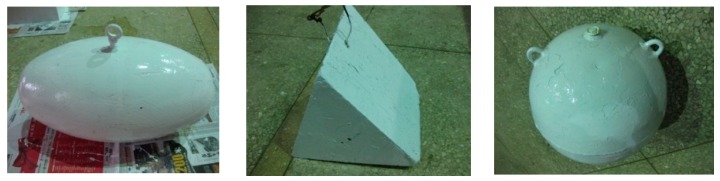
Target models.

**Figure 11:**
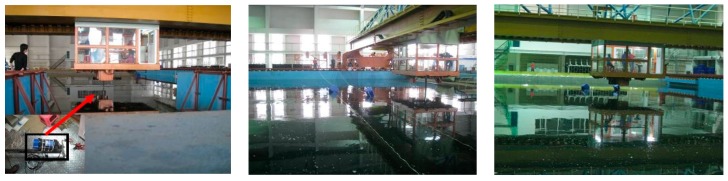
Experiment environment.

In the experiment, the moving target consisted of three types of targets (shown in Figure 10). A trailer was selected as the platform on which FLS was fixed. Due to the limitation of the tank length, trailer and FLS remained static during the whole experiment, and the targets are dragged by ropes on both sides of the tank. Parts of test scene are shown in Figure 11. For each image sequence, the number of particles was set to 300 in per frame, that is, 300 candidates were collected around the position of the target in the previous frame. The same image-processing algorithms were used to compare the accuracy gained by the proposed algorithm with ones gained by other algorithms, and the parameters in the image-processing algorithm were set as the same value.

#### 5.1.1. Comparative Experiments of Tracking Methods

**Figure 12:**
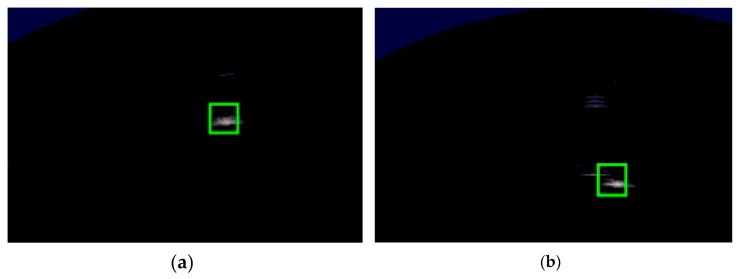
Tracking results obtained by presented method in (**a**) (p + 1)th image and (**b**) (p + 2)th image.

**Figure 13:**
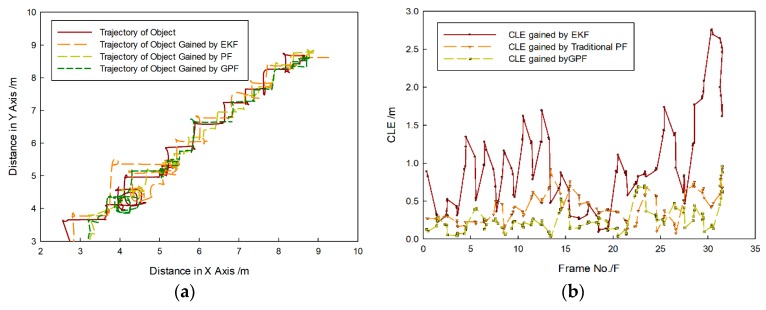
Comparison results obtained by different methods: (**a**) tracking trajectory gained by each method and (**b**) center-position-error (CLE) curve gained by each method.

In order to analyze tracking performance, tracking experiments of a single target were carried out (shown in Figure 12), and results are shown in Figure 13.

Figure 12 shows that the moving target was close to the FLS from far and near, and the target region was quite changeable, but the proposed method could effectively track the target. During the entire tracking process, some influence existed, such as fluid resistance and the drag speed of the rope, so the moving direction of the target often suddenly changed. Then, its trajectory did not obviously appear with regular motion, as shown in Figure 13a, but target can be tracked by each method. In comparison with each other, the tracking trajectory obtained by the proposed method was closer to the real trajectory. The EKF is the approximation of the nonlinear non-Gaussian motion state. Its tracking accuracy is sensitive to the target-motion state, and cumulative error appears in the tracking process, so the CLE gained by EKF was bigger than that gained by other methods, and it had a trend of divergence, as shown in Figure 13b. Instead, PF and the proposed method are nonlinear filtering methods based on Monte Carlo simulations, so the CLE gained by PF and proposed method were in a small stable interval, and tracking results were more accurate. For the proposed method, it was not necessary to input strong prior knowledge into the state equation and measurement equation, so variance of the target movement had less influence on the tracking result. Thus, the CLE gained by the proposed method was smaller, and tracking was more accurate.

#### 5.1.2. Fusion-Strategy Experiments

In the experiments, targets kept moving along different paths of motion, and only parts of the results gained under the existence of crossing and noncrossing trajectories are shown due to the limitations of this paper.

**Figure 14:**
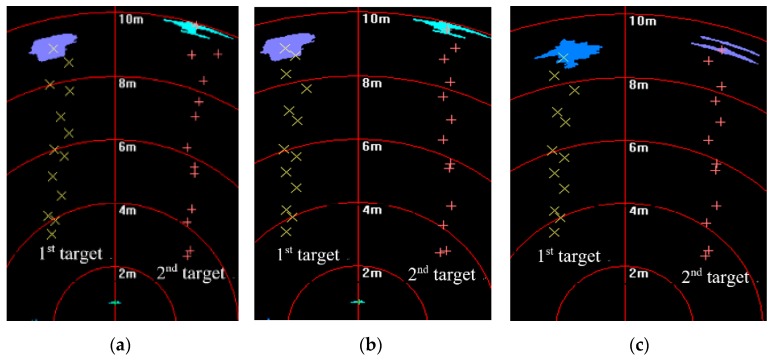
Tracking results based on different fusion strategies gained by: (**a**) multiplicative fusion (MF); (**b**) weighted fusion (WF), and (**c**) proposed method.

**Figure 15:**
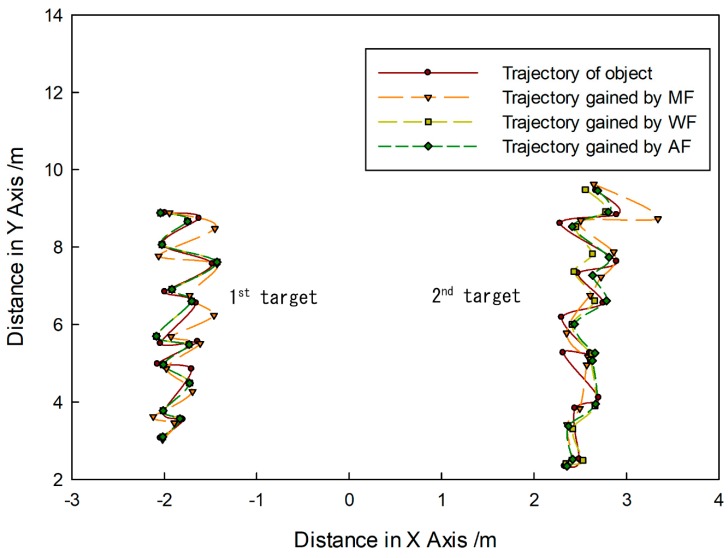
Tracking trajectory gained by different fusion methods.

**Figure 16:**
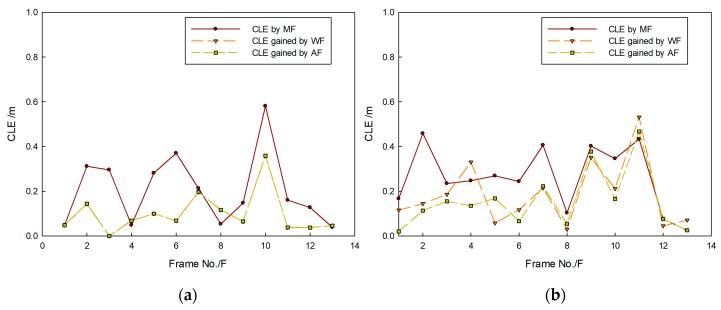
CLE gained by different fusion methods: results of (**a**) first target and (**b**) second target.

Figure 14 shows that targets moved in the same direction and they were close to the FLS from far and near. In the whole moving phase of the targets, targets could be caught by three fusion methods. In Figure 15, it is shown that target trajectories had unsteady fluctuation, which led to the larger tracking deviation gained by MF. In this situation, the fusion algorithm was selected by feature analysis in the proposed method. As feature clues were degenerated, WF was used to calculate the likelihood function, so trajectories gained were closer to those gained by WF. In Figure 16, it shown that all CLE curves were affected by the unsteady motion of targets, and they had the same trend. By contrast, the CLE curve of the first target gained by the proposed method was almost coincident with that gained by WF, but the CLE of the second target gained by the proposed method is smallest, showing that the proposed method could track targets more accurately.

**Figure 17:**
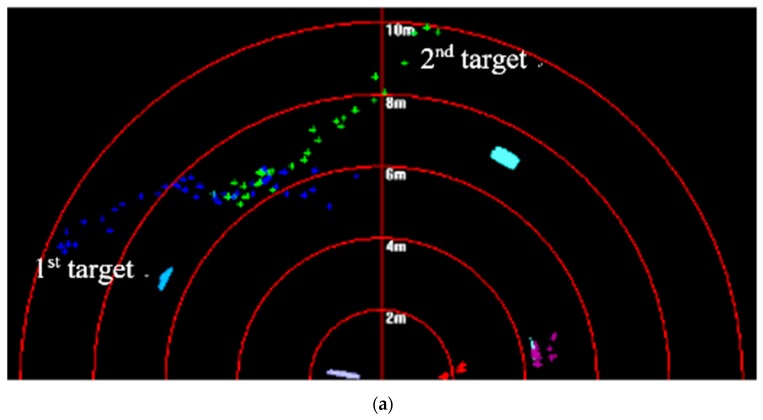

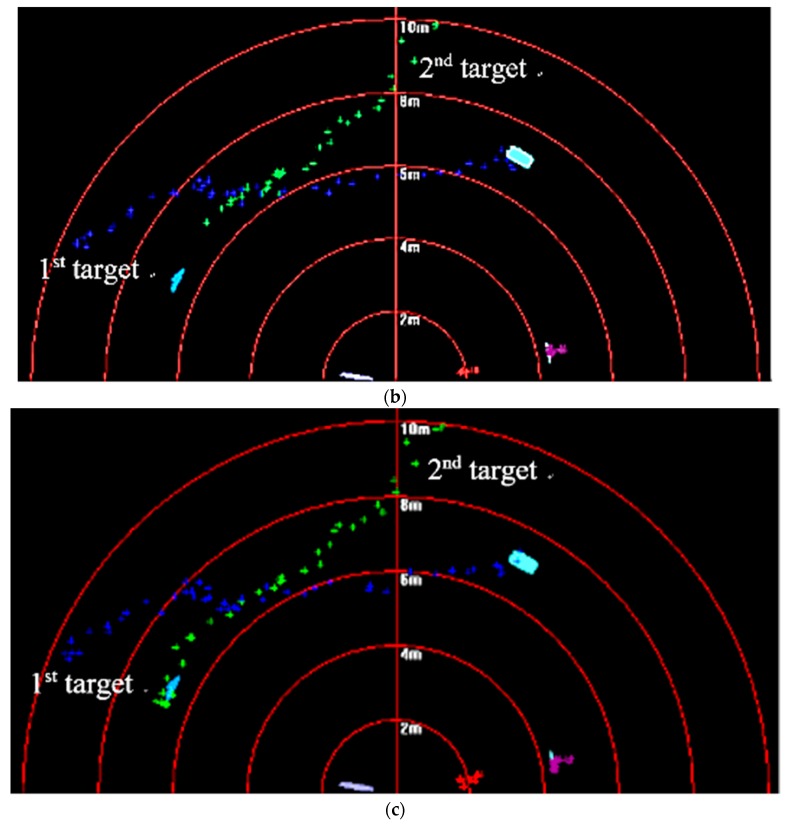
Tracking results based on different fusion strategies gained by (**a**) MF; (**b**) WF; and (**c**) proposed method.

**Figure 18:**
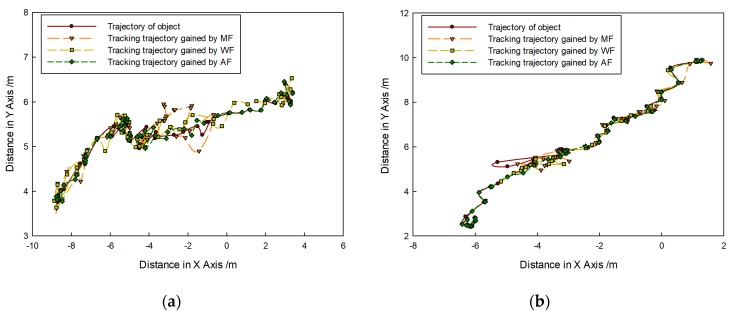
Tracking trajectory gained by different fusion methods of (**a**) first target and (**b**) second target.

**Figure 19:**
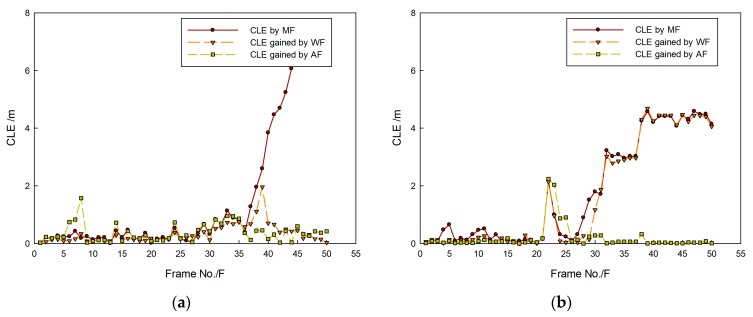
CLE gained by different fusion methods: results of (**a**) first target and (**b**) second target.

Figure 17 shows that targets moved in the opposite direction, and their trajectories intersect. Targets could be caught by three fusion methods before they met each other. After they left the intersection point, not all targets could be caught by MF. Using WF, the first target could be caught, but the second target was lost. By contrast, the proposed method was successful in consecutively tracking targets. In Figure 18, it is shown that all predicted target trajectories were close to the real trajectory before they met each other. By contrast, trajectory deviation gained by MF is larger, but they are similar with those gained by WF and proposed method. As targets met each other, all predicted trajectories were affected. After they left the intersection point, the predicted trajectory gained by the MF gradually strayed away from the real target position, which indicated that target tracking was a failure. Results gained by WF showed that the tracking of the second target was a failure. Although the first target was caught, its predicted trajectory wildly fluctuated, which led to a decrease in tracking accuracy. However, predicted trajectories gained by proposed method shortly fluctuated, which were in the controlled range; thus, target tracking remained continuous. In Figure 19, it is shown that, in comparison with the CLE divergence gained by other methods, the CLE gained by the proposed method remained a low stable during the tracking process, so the proposed method was more robust and it could maintain the smoothness of the tracking curve faster in the interference environment.

### 5.2. Sea Trial

**Figure 20:**
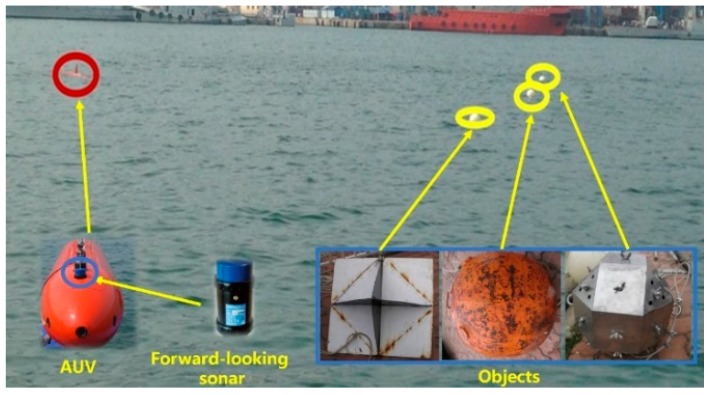
Sea-trial scene.

To further evaluate the proposed method, a series of trials were carried out in the sea, where depth of water was 10 m. An AUV named cShark was used as the moving platform, which was developed by Harbin Engineering University. cShark is about 5.5 m long, 0.8 m wide, and the redundancy of its actuators provides important functionalities, such as accurate perception and fine motion. Target size was less than 1 ×1 m, and they were located at 3 m underwater. Float balls were mounted on top of the targets, and ballasts were fixed on the bottom of targets, then, targets were levitated in the water. Targets were dragged by ropes and current, and their velocities were about 0.5–1 m/s. The AUV was kept running at the same depth as the targets, and the moving targets were tracked online by FLS. AUV speed was about 0.5 m/s. The sea-trial scene is shown in Figure 20.

#### 5.2.1. Acoustic-Vision-Based Processing Framework

**Figure 21:**
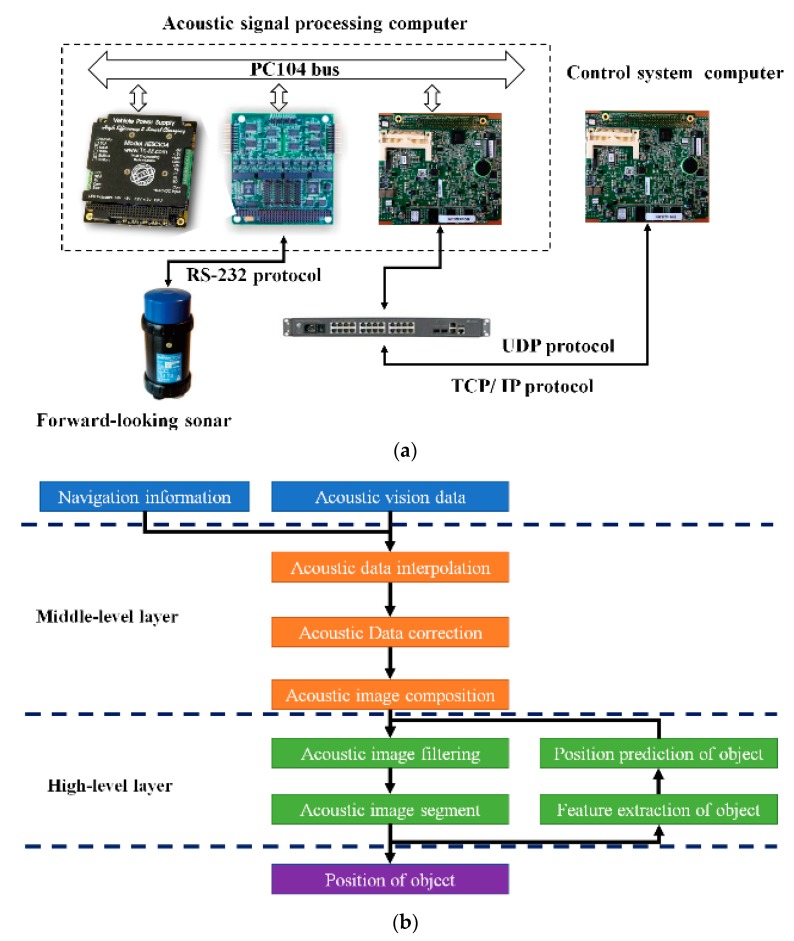
Acoustic-vision-based framework: (**a**) hardware architecture and (**b**) software architecture.

The hardware architecture comprises two parts that are shown in Figure 21a. One is an acoustic signal-processing computer, which is where the sonar-controller software and acoustic-image-processing software are run, and it passes the predicted measurements to the controller computer through a high-speed internal network. The second part is an FLS, which was facing front, and its detection range was set to 50 m. On the basis of Marr visual theory, software architecture was developed in the C language and included two parts, the middle- and high-level layers (shown in Figure 21b). The middle layer is for image preprocessing, such as image-data-interpolation processing, and acoustic images are formed on the basis of echo data collected at different times. The high-level layer is the ultimate implementation part. Acoustic images are processed, and target regions are gained. The possible region is predicted by the GPF, and the number of particles was set to 200. Results are submitted to the control system for planning the AUV navigation route, and are also used to determine the image-processing region in the next frame.

#### 5.2.2. Target-Tracking Test under Noncrossing-Movement Condition

**Figure 22:**
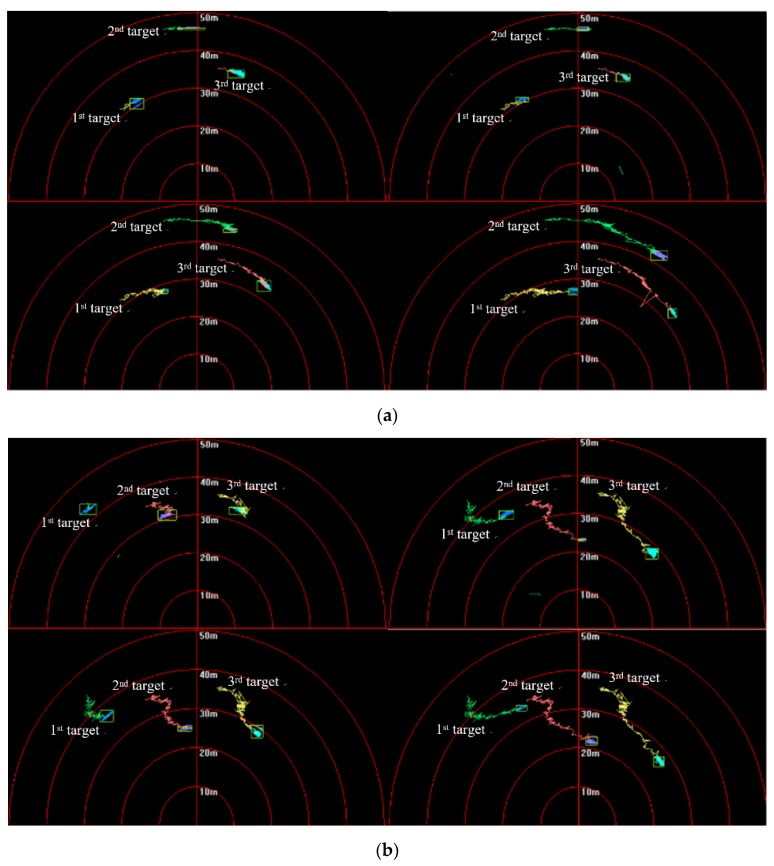
Target-tracking results in: (**a**) first and (**b**) second sequence of forward-looking-sonar (FLS) images.

In Figure 22, it is shown that targets move in the same direction, and the relative position varied with the movement of targets and the AUV. Then, reflection surfaces were changeable, so target regions in the FLS images were obviously gradually different. In the whole moving phase of the targets, the real trajectory was not smooth, and a situation of sudden change existed. Despite all this, continuous and stable target tracking was maintaining from the beginning by the proposed method.

**Figure 23:**
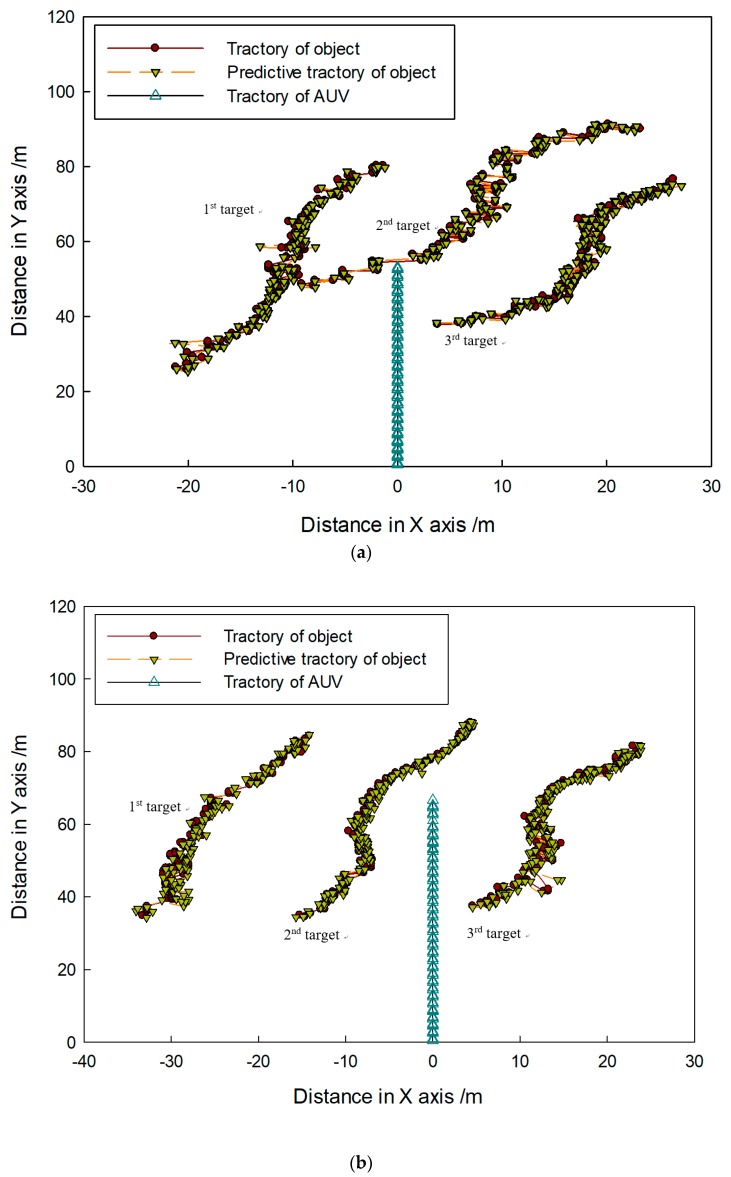
Target-tracking trajectory in (**a**) first and (**b**) second sequence of FLS images.

Figure 23 shows that rope and current disturbance were more serious those that in the tank experiment, so variation of real trajectories was sharper, and it is seemed that they sometimes moved by leaps and bounds. However, targets were still caught by proposed method, which maintained stable target tracking, and gained trajectories were close to the real ones.

**Figure 24:**
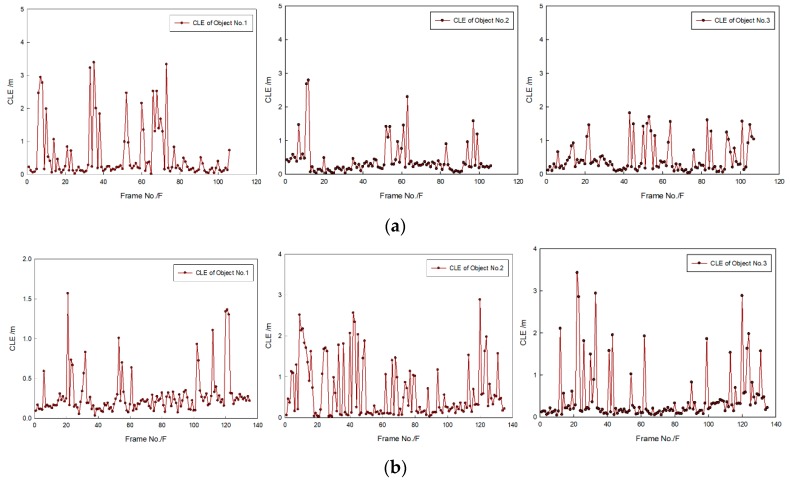
CLE gained by proposed method: results in (**a**) first and (**b**) second sequence of FLS images.

Figure 24 shows that, because of the influence of the current and AUV movement in the sea test, tracking error was larger than that obtained in the tank experiment, so CLE curves swung significantly and violently. In general, most CLEs obtained by the proposed method remained lower, which indicates that the method could be used to maintain the target tracking under an unstable condition of target movement.

#### 5.2.3. Target-Tracking Test under Crossing-Movement Condition

**Figure 25:**
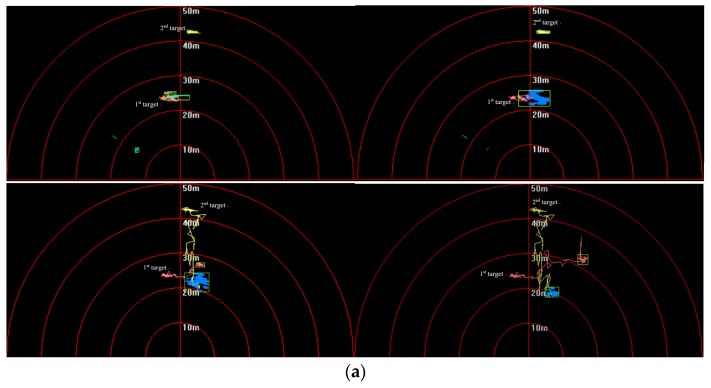

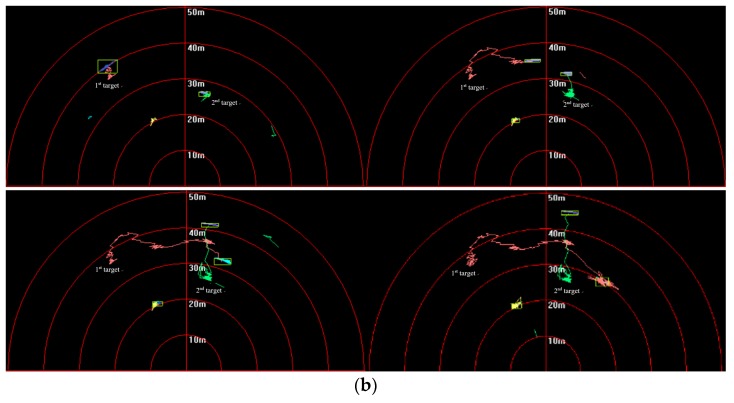
Targets-tracking results in (**a**) first and (**b**) second sequence of FLS images.

**Figure 26:**
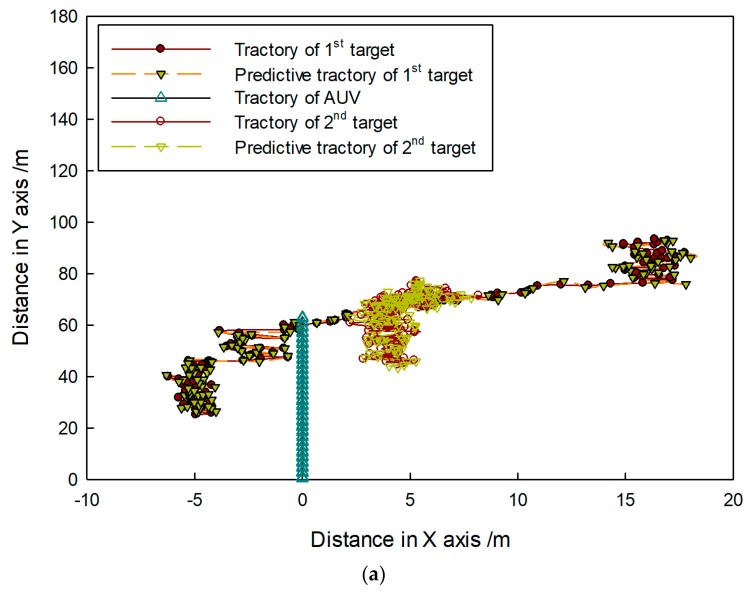

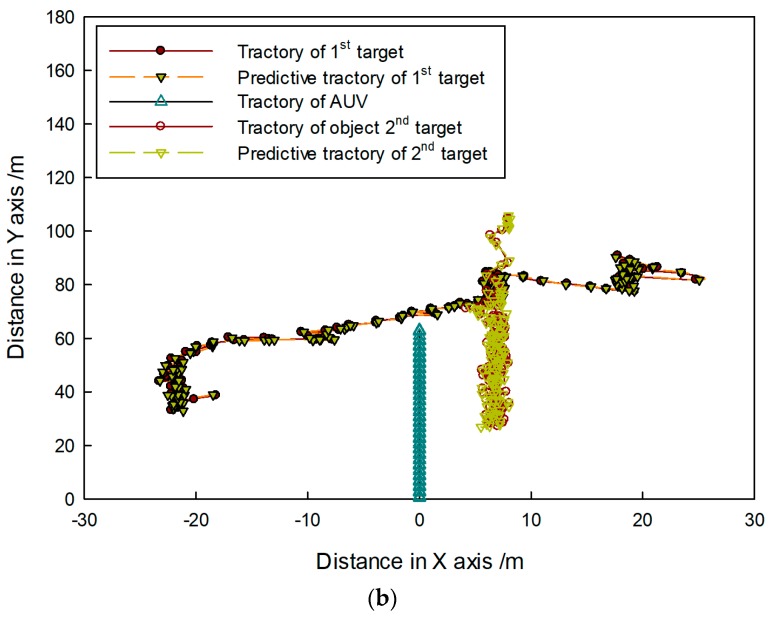
Target-tracking trajectory in (**a**) first and (**b**) second sequence of FLS images.

**Figure 27:**
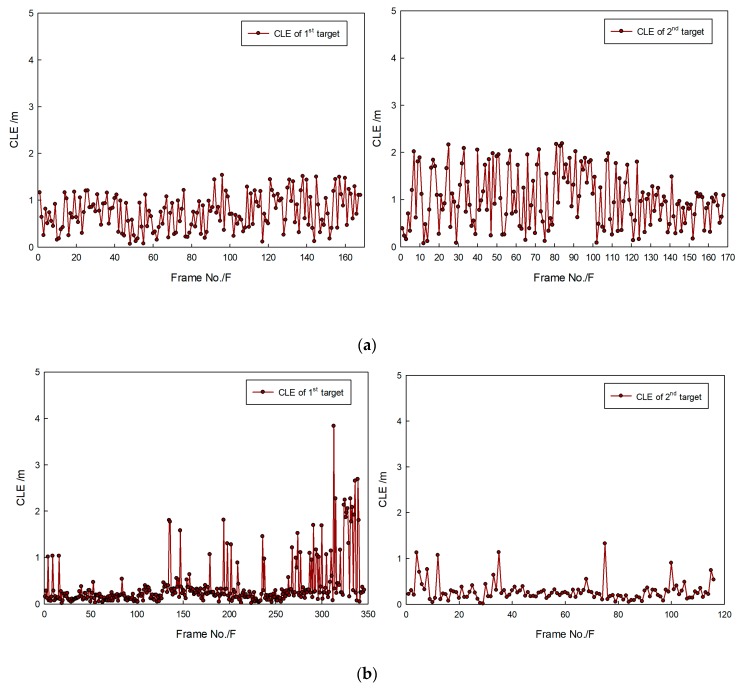
CLE gained by proposed method: results in (**a**) first and (**b**) second sequence of FLS images.

In the sea trial, it was hard to make more than two targets move under the existence of a crossing path. Therefore, tracking problems of two kinds of targets are still considered of which trajectories intersect. Results are shown in Figure 25, Figure 26 and Figure 27.

In Figure 25, targets are shown to move in different directions, and some features such as area, length, and shape obviously changed with the movement. In Figure 26, it shown that target trajectories were not smooth. As they met each other, the predicted trajectories were more cluttered for interference. The proposed method was not affected by them, and it could accurately lock onto the target position, so tracking status remained continuous and steady.

Figure 27a shows that the real trajectory of the second target often suddenly changed, of which the variable range is larger. As a result, tracking accuracy decreased, and the CLE curves of the second target swung significantly and violently. Overall, however, the average CLE values of the first and second targets were about 1 m. Figure 27b shows that some abrupt change points existed because of the influence of target movement in leaps and bounds. In general, most target CLE remained lower most of the time, which indicates that the method is effective for target tracking.

## 6. Conclusions

In this paper, we proposed an AUV underwater-target-tracking framework based on acoustic images. An acoustic image received from an imaging sonar is unstable due to ultrasonic waves. Hence, it is difficult to continuously detect and track targets. To solve this problem, a GRNN was designed to select target features, and the effectiveness of the feature candidates in a series of images was evaluated. Furthermore, an adaptive fusion was used to establish the observation model, and the improved GPF was adopted to track moving targets. The tank and sea tests illustrated that this method is flexible in tracking moving targets in cluttered unknown environments, and it can solve the target-tracking problem under the crossed-path condition. The next stage of this work is to use the method presented in [41] and [42] to enhance the proposed fusion approach and classification results, and to apply this algorithm in more complicated ocean environments, where time-variable ocean currents and dynamic targets exist.

## Figures and Tables

**Table 1:** Sonar specifications.

Parameter	Operating Frequency	Horizontal Beam Width	Vertical Beam Width	Maximum Range	Range Resolution	Scan Size	Weight
Low-frequency model	325 KHz	3.0°	20°	300 m	about 15 m	0°–360°	3 kg in air, 1.4 kg in water
High-frequency model	675 KHz	1.5°	40°	100 m			

**Table 2:** (**a**) Basic features of regions. (**b**) Contrast features of regions. (**c**) Shape moment features of regions. (**d**) Moment-invariant features of regions. (**e**) Statistical-texture features of regions.

No.	Function
	(**a**)
1	Area $A_{0}=N^{o}$
2	Perimeter length $P_{0}=N^{eo}$
3	Mean intensity $\;\;{\bar{I}}_{0}=\left[\sum_{i=1}^{m}\sum_{j=1}^{n}f\left(i,j\right)\right]/N^{o}$ $\;\mathrm{and}\;p\left(i,j\right)\in R_{k}$
4	Intensity standard deviation $\sigma_{0}=\left[\sum_{i=1}^{m}\sum_{j=1}^{n}{\left[f\left(i,j\right)-{\bar{I}}_{0}\right]}^{2}\right]/N^{o}$ $\mathrm{and}\;p\left(i,j\right)\in R_{k}$
5	Compactness $O_{0}=4\pi A_{0}/{\left(P_{0}\right)}^{2}$
6	Background mean ${\bar{B}}_{0}=\left[\sum_{i=1}^{m}\sum_{j=1}^{n}f\left(i,j\right)\right]/N^{b}\;\mathrm{and}\;p\left(i,j\right)\notin R_{k}$
	(**b**)
7	$C_{0}^{1}={\bar{I}}_{0}-{\bar{B}}_{0}$
8	$C_{0}^{2}={\bar{I}}_{0}/{\bar{B}}_{0}$
9	$C_{0}^{3}=\left({\bar{I}}_{0}-{\bar{B}}_{0}\right)/\left({\bar{I}}_{0}+{\bar{B}}_{0}\right)$
	(**c**)
10	$SM_{1}={\left[\left(\sum_{t=1}^{P_{0}}{\left[D_{e}^{o}\left(i\right)-\sum_{t=1}^{P_{0}}D_{e}^{o}\left(i\right)/\;P_{0}\right]}^{2}\right)/P_{0}\right]}^{1/2}/\sum_{t=1}^{P_{0}}D_{e}^{o}\left(i\right)/\;P_{0}$
11	$SM_{2}={\left[\left(\sum_{t=1}^{P_{0}}{\left[D_{e}^{o}\left(i\right)-\sum_{t=1}^{P_{0}}D_{e}^{o}\left(i\right)/\;P_{0}\right]}^{3}\right)/P_{0}\right]}^{1/3}/\sum_{t=1}^{P_{0}}D_{e}^{o}\left(i\right)/\;P_{0}$
12	$SM_{3}={\left[\left(\sum_{t=1}^{P_{0}}{\left[D_{e}^{o}\left(i\right)-\sum_{t=1}^{P_{0}}D_{e}^{o}\left(i\right)/\;P_{0}\right]}^{4}\right)/P_{0}\right]}^{1/4}/\sum_{t=1}^{P_{0}}D_{e}^{o}\left(i\right)/\;P_{0}$
13	$SM_{4}=SM_{3}-SM_{1}$
	(**d**)
14	$M_{1}=\eta_{20}+\eta_{02}$
15	$M_{2}={\left(\eta_{20}-\eta_{02}\right)}^{2}+4\eta_{11}^{2}$
16	$M_{3}={\left(\eta_{30}-3\eta_{12}\right)}^{2}+{\left(3\eta_{21}-\eta_{03}\right)}^{2}$
17	$M_{4}={\left(\eta_{30}+\eta_{12}\right)}^{2}+{\left(\eta_{21}+\eta_{03}\right)}^{2}$
18	$M_{5}=\left(\eta_{30}-3\eta_{12}\right)\left(\eta_{30}+\eta_{12}\right)\left[{\left(\eta_{30}+\eta_{12}\right)}^{2}-3{\left(\eta_{21}+\eta_{03}\right)}^{2}\right]+\left(3\eta_{21}-\eta_{03}\right)\left(\eta_{21}+\eta_{03}\right)$ $\left[3{\left(\eta_{30}+\eta_{12}\right)}^{2}-{\left(\eta_{21}+\eta_{03}\right)}^{2}\right]$
19	$M_{6}=\left(\eta_{20}-\eta_{02}\right)\left[{\left(\eta_{30}+\eta_{12}\right)}^{2}-{\left(\eta_{21}+\eta_{03}\right)}^{2}\right]+4\eta_{11}\left(\eta_{30}+\eta_{12}\right)\left(\eta_{21}+\eta_{03}\right)$
20	$M_{7}=\left(3\eta_{21}-\eta_{03}\right)\left(\eta_{30}+\eta_{12}\right)\left[{\left(\eta_{30}+\eta_{12}\right)}^{2}-3{\left(\eta_{12}+\eta_{03}\right)}^{2}\right]+\left(\eta_{30}-3\eta_{12}\right)\left(\eta_{21}+\eta_{03}\right)$ $\left[3{\left(\eta_{30}+\eta_{12}\right)}^{2}-{\left(\eta_{21}+\eta_{03}\right)}^{2}\right]$
	(**e**)
21	Inertia $M_{1}^{co}=\sum_{i=0}^{s-1}\sum_{j=0}^{s-1}{\left(i-j\right)}^{2}h\left(i,j\right)$
22	Entropy $M_{2}^{co}=-\sum_{i=0}^{s-1}\sum_{j=0}^{s-1}h\left(i,j\right)\ln h\left(i,j\right)$
23	Angular second moment $M_{3}^{co}=\sum_{i=0}^{s-1}\sum_{j=0}^{s-1}h{\left(i,j\right)}^{2}$
24	Inverse difference moment $M_{4}^{co}=\sum_{i=0}^{s-1}\sum_{j=0}^{s-1}\{h\left(i,j\right)/[1+\left(i-j{)}^{2}\right]\}$
25	Correlation
26	Variance $M_{6}^{co}=\sum_{i=0}^{s-1}\sum_{j=0}^{s-1}{\left(i-\mu_{x}\right)}^{2}h\left(i,j\right)$
27	Sum average $\;M_{7}^{co}=\sum_{k=2}^{2s}k\left[\sum_{i=0}^{s-1}\sum_{j=0}^{s-1}h\left(i,j\right)\right]$,i+j=k
28	Sum entropy $M_{8}^{co}=-\sum_{k=2}^{2s-2}\left[\sum_{i=0}^{s-1}\sum_{j=0}^{s-1}h\left(i,j\right)\right]\ln[\sum_{i=0}^{s-1}\sum_{j=0}^{s-1}h\left(i,j\right)],\;i+j=k$
29	Sum variance $M_{9}^{co}=\sum_{k=2}^{2s-2}{\left(k-M_{7}^{co}\right)}^{2}\left[\sum_{i=0}^{s-1}\sum_{j=0}^{s-1}h\left(i,j\right)\right],\;i+j=k$
30	Difference entropy $M_{10}^{co}=-\sum_{k=0}^{s-1}\left[\sum_{i=0}^{s-1}\sum_{j=0}^{s-1}h\left(i,j\right)\right]\ln[\sum_{i=0}^{s-1}\sum_{j=0}^{s-1}h\left(i,j\right)],\left|i-j\right|=k$

**Table 3:** Sequential-forward-selection (SBS) procedure.

Algorithm of SBS	
1	Start with the full set $Y_{0}=X$
2	Remove the worst feature $x^{-}=\arg\max J\left(Y_{k}-x\right)$, $x\in Y_{k}$
3	Update $Y_{k+1}=Y_{k}-x^{-}$; k=k+1
4	Go to 2

**Table 4:** Sequential-backward-selection (SFS) procedure.

Algorithm of SFS	
1	Start with the empty set $Y_{0}=\left\{\emptyset \right\}$
2	Select the next best feature $x^{+}=\arg\max J\left(Y_{k}+x\right)$, $x\in Y_{k}$
3	Update $Y_{k+1}=Y_{k}+x^{+}$; k=k+1
4	Go to 2

**Table 5:** Experiment situation.

No.	Description
1	Only first target moves.
2	Only second target moves.
3	Only third target moves.
4	Only fourth target moves.
5	First and fourth targets move together in the same direction.
6	Second and fourth targets move together in the same direction.
7	Fourth and fifth targets move together in the same direction.
8	Third and fourth targets move together in the opposite direction, and their trajectory is crossed.
9	Third and fifth targets move together in the opposite direction, and their trajectory is crossed.
10	First and second targets move together in the opposite direction, and their trajectory is crossed.
11	Second and third targets move together in the opposite direction, and their trajectory is crossed.
12	Second, third, and fourth target moves together in the same direction.
13	Second, third, and fourth targets move together in the opposite direction.
14	Second target does not move.

**Table 6:** Feature intervals.

Interval No	B	C	D	E	F	G
Feature No	1–5	6–10	11–15	16–20	21–25	26–30

**Table 7:** Selected features.

Feature Order Gained by SFS	1	2	3	4	5
Feature No.	20	17	3	6	24

**Table 8:** Computation procedure of $a_{i}.$

$\mathrm{Algorithm}\;\mathrm{of}\;a_{i}\mathrm{Calculation}$	
1	Calculate value ${\bar{f}}_{else,i}$, which is written as: ${\bar{f}}_{else,i}=\left(\sum_{j=0}^{n}1/\Delta_{j}\right)/m,j\neq i$
2	Design fuzzy controller to translate $1/\Delta_{j}$ and ${\bar{f}}_{else,i}$ to fuzzy domain; fuzzy-rule table is shown in Table 9.
3	Input $1/\Delta_{j}$ and ${\bar{f}}_{else,i}$ into the fuzzy controller, and obtain fuzzy output $b_{i}$ of *i*^th^ feature.
4	Calculate weighting coefficients of each feature $a_{i}$, which is written as: $a_{i}=b_{i}/\Sigma_{i=1}^{m}b_{i}$

**Table 9:** Fuzzy rule list of $a_{i}$.

${\bar{f}}_{else,i}$	$1/\Delta_{j}$				
	NB	NS	ZE	PS	PB
NB	3	4	4	5	5
NS	2	3	4	4	5
ZE	2	2	3	4	4
PS	1	2	2	3	4
PB	1	1	2	2	3
